# Association between Number of Teeth and Eating out of Home: A 2019 Statistical Survey of the Japanese Representative Population

**DOI:** 10.3390/nu16132102

**Published:** 2024-07-01

**Authors:** Anna Kinugawa, Takafumi Yamamoto, Taro Kusama, Kenji Takeuchi, Ken Osaka

**Affiliations:** 1 Department of International and Community Oral Health, Graduate School of Dentistry, Tohoku University, 4-1, Seiryo-machi, Aoba-ku, Sendai 980-8575, Miyagi, Japan; kinugawa.annna.r3@dc.tohoku.ac.jp (A.K.); taro.kusama.a2@tohoku.ac.jp (T.K.); kenji.takeuchi.c4@tohoku.ac.jp (K.T.); ken.osaka.e5@tohoku.ac.jp (K.O.); 2 Preventive Dentistry, Hokkaido University Hospital, Kita 13 jo Nishi 7 Chome, Kita-ku, Sapporo 060-8586, Hokkaido, Japan; 3 Department of Health Promotion, National Institute of Public Health, 2-Chome-3-6 Minami, Wako 351-0104, Saitama, Japan; 4 Division of Statistics and Data Science, Liaison Center for Innovative Dentistry, Graduate School of Dentistry, Tohoku University, 4-1, Seiryo-machi, Aoba-ku, Sendai 980-8575, Miyagi, Japan

**Keywords:** eating out, oral health, older population

## Abstract

Eating out of home (EOH), with its diverse food options, can benefit those with difficulty preparing their meals, especially older adults. Oral health status may be a determinant of EOH, as food accessibility is influenced by oral health, but this association remains unclear. This cross-sectional study used merged data from two national statistical surveys conducted in 2019. Participants were individuals aged ≥ 65 years who responded to both surveys. The frequency of EOH (<once/week or ≥once/week) was the dependent variable. The number of teeth was used as the independent variable (≥20, 10–19, 1–9, and 0). Prevalence ratios (PRs) and 95% confidence intervals (CIs) were calculated using multivariate Poisson regression analysis to identify the association between EOH and the number of teeth, adjusting for possible confounders. We analyzed 2164 participants (mean age = 74.0, women 52.4%). Of these, 456 (21.1%) participants were EOH ≥ once/week; 1142 (52.8%) participants had ≥20 teeth. Compared to those with ≥20 teeth, those with <20 teeth had a lower prevalence of EOH ≥ once/week (10–19: PR = 0.89, 95% CI = 0.72–1.09, 1–9: PR = 0.67, 95% CI = 0.51–0.89, and 0: PR = 0.53, 95% CI = 0.36–0.77, respectively). We observed an association between fewer teeth and a lower frequency of EOH.

## 1. Introduction

Malnutrition is a critical concern among older adults as it is linked to various diseases, such as functional disability [1] and frailty [2]. One strategy to prevent malnutrition is to ensure food accessibility [3]. An opportunity to maintain food accessibility is eating out of home (EOH) [4]. Although the definition of EOH varies [5], it is commonly understood as consuming meals prepared outside, such as at restaurants, and this behavior is increasing worldwide [6,7]. EOH encompasses both dietary intake behaviors and going out, so the reasons why older adults engage in EOH vary. For example, when older adults live alone, they may lose the motivation to cook or eat regularly [8]. This lack of motivation could increase EOH [9]. Additionally, for older adults, EOH is considered a form of social interaction that includes conversation during meals [10]. In previous studies, EOH has been viewed as a health-risk behavior owing to increasing opportunities for high-calorie food intake [11] and nutritional imbalances in food intake [5]. However, studies on older adults have shown that those who ate prepared meals had better diet quality compared to those who did not receive meals from senior nutrition programs [5,12]. It has been found that older adults with poor cooking skills also have poor diet quality [13], and the meals provided through EOH may help ensure better diet quality for them. Moreover, EOH can balance convenience and nutrition if restaurants offer healthier meals. Indeed, encouraging healthy food choices during EOH is being increasingly recognized as a necessary public health intervention [11,14]. Intervention studies on EOH have observed an increase in consumers choosing healthier foods when eating out [15,16]. Therefore, EOH may be a critical means of accessing food, especially for older adults who cannot cook.

Oral health may be associated with EOH from the perspective of food mastication. For example, it has been suggested that older adults with fewer teeth consume fewer healthy foods such as meat or vegetables [17,18,19]. Previous studies have shown that having fewer teeth can restrict food choices [20]. Additionally, having fewer teeth can impair authentic appearance, leading to reduced going out [21]. Therefore, it is possible that having fewer teeth may also restrict EOH among older adults. Having few teeth is not only a risk factor for malnutrition [22] but also potentially associated with reduced food accessibility. If oral health impacts food accessibility, then nutrition policies for older adults should be incorporated with oral health policies. Therefore, this study has significant public health implications. However, only a few studies have examined this relationship. Alternatively, having fewer teeth may be related to EOH as a proxy for socioeconomic status (SES) because low SES can lead to having fewer teeth [23], and EOH is more expensive than eating at home in most countries [5].

Based on previous studies, we propose a theoretical framework where the number of teeth affects EOH frequency among older adults. This framework suggests that having fewer teeth leads to difficulties in mastication, reducing the frequency of EOH due to decreased motivation and physical capability to eat out. Given the cross-sectional nature of our study, we explore associations rather than causations, providing a basis for future longitudinal research to investigate these complex interactions. This study aimed to observe the association between the number of teeth and frequency of EOH among community-dwelling older adults.

## 2. Methods

### 2.1. Setting and Participants

This cross-sectional study was based on secondary analysis of data obtained from the 2019 Comprehensive Survey of Living Conditions (CSLC) and the 2019 National Health and Nutrition Survey (NHNS), a nationally representative survey conducted by the Japanese Ministry of Health, Labour and Welfare [24,25]. Details of the CSLC and NHNS have been previously described [26,27].

The 2019 CSLC was conducted in 5530 districts using stratified random sampling from districts across the country and included all households living in these districts (approximately 300,000 households or 720,000 individuals) in June. The response rate for the 2019 CSLC was 72.5% (218,332 of 301,334 households). The 2019 NHNS was conducted in 296 districts selected using stratified random sampling methods from the 5530 districts set in the 2019 CSLC and included all households living in those districts in November. The response rate for the 2019 NHNS was 63.5% (2836 out of 4465 households). In both the CSLC and NHNS, if there were multiple individuals in one household, questionnaires were mailed to each individual. Participants aged 65 years and older who responded to both the CSLC and NHNS were included. We excluded the participants aged < 65 years, hospitalized patients, nursing home residents, and those with missing data on subjective symptoms and hospital visits.

### 2.2. Dependent Variable

We used the frequency of EOH from the NHNS questionnaire as the dependent variable, which was assessed using the following question: “How often do you eat out (eating at the restaurant)?” There were seven choices: “two times or more per day”, “once per day”, “four to six times per week”, “two to three times per week”, “once per week”, “less than once per week”, and “never”. Participants were categorized into the following two categories: “less than once per week” and “once or more per week” [28].

### 2.3. Independent Variable

The number of teeth in the NHNS questionnaire was used as the independent variable. The number of teeth was assessed using the question “How many teeth do you have, excluding wisdom teeth, dentures, dental bridges, and implants? Generally, we have 28 teeth except wisdom teeth, but there are people with more than 28 or less than 28 teeth”. The reliability of self-reported number of teeth was confirmed in a previous study [29]. Participants in this study responded with continuous values ranging from 0 to 32. We categorized the response as follows: no teeth, 1–9 teeth, 10–19 teeth, and ≥20 teeth.

### 2.4. Covariates

We included several variables as covariates based on previous studies and clinical knowledge [28,30]: age (65–69, 70–74, 75–79, 80–84, and ≥85 years), sex (men and women), smoking status (never, quit, and current), drinking habits (never, quit, and current), educational attainment (≤9, 10–12, and ≥13 years), living arrangement (living alone and living with others), marital status (married and not married), working status (working and not working), psychological distress (low and high psychological distress), medical institution visits (no and yes), subjective health status (good, normal, and bad), healthy food intake (no and yes), and social participation (no and yes). Age and sex were used as demographic variables. Smoking status and drinking habits were used as health risk behavior variables because they are risk factors for EOH behavior [31] and the number of teeth [32]. Educational attainment and working status were used as SES. Previous studies have suggested that the number of remaining teeth was associated with education [33] and their working status [34]. Additionally, EOH behavior is strongly related to their economic status [5]. Therefore, we included educational attainment and working status in our model. Living arrangement, marital status, and social participation were used as social factors. EOH behavior is considered to be one of the social activities [5] and a risk factor for the number of teeth [21]. Psychological distress, subjective health status, healthy food intake, and medical visits were used as health statuses because EOH behavior is considered to be affected by their health status and health awareness [5]. Oral health status is also associated with overall health status [35]. Psychological distress was assessed using the Japanese version of the 6-item Kessler Scale (K6) [36,37]. We divided participants into those with low psychological distress (K6 score < 5) and high psychological distress (K6 score ≥ 5) based on the cutoff value of the previous study [38]. We described the details of the covariates in Supplementary Table S1. The covariates from the CSLC questionnaire included age, sex, educational attainment, living arrangement, marital status, working status, psychological distress, medical institution visits, subjective health status, and healthy food intake, whereas those from the NHNS questionnaire included smoking status, drinking habits, and social participation.

### 2.5. Statistical Analysis

Poisson regression was performed to investigate the association between the number of teeth and frequency of EOH. We calculated the prevalence ratio (PR) and 95% confidence intervals (95% CIs) for three models: Model 1 was the crude model, Model 2 was adjusted for age and sex, and Model 3 was adjusted for all covariates. Additionally, we conducted the trend of the association using the number of teeth as a continuous variable. In this study, 30% of participants had missing values for some variables. To reduce the bias introduced by missing data and improve the validity of the estimations, we implemented multiple imputation [39]. All missing values were imputed based on multiple imputation using chained equations and 20 imputed datasets were created. Estimates obtained from each imputed dataset were combined using Rubin’s rule [40]. We conducted a complete case analysis as a sensitivity analysis to confirm the consistency of the results of multiple imputation data. Additionally, two sensitivity analyses were performed. First, we explored the association between the number of teeth and the frequency of EOH according to sex. Previous studies have reported that there were differences not only in the number of teeth, but also in eating behavior between men and women [5,41]. Additionally, we included an interaction term between number of teeth and sex in the model and conducted a Poisson regression analysis. Second, to control the potential impact of restaurant density in their residential area on EOH behavior, we conducted a sensitivity analysis that included city-level variables in Model 3. The population of the municipality was divided into three categories: metropolitan areas, cities with populations of 150,000 or more, and cities with populations of less than 150,000, including towns and villages. All analyses were performed using STATA software (version 18.0; Stata Corp., College Station, TX, USA). The level of significance was set at *p* < 0.05.

### 2.6. Ethical Approval

This study was approved by the Research Ethics Committee of the National Institute of Public Health (No. 12430).

## 3. Results

**Figure 1:**
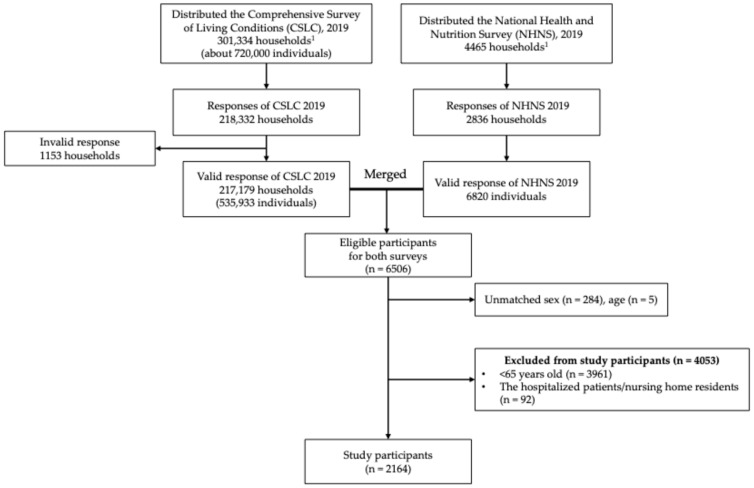
Flow chart of the study participants. Note: CSLC, Comprehensive Survey of Living Conditions; NHNS, National Health and Nutrition Survey. ^1^ Both of the two surveys targeted the household.

Figure 1 shows the flowchart of the study participants. The number of participants in the merged CSLC and NHNS datasets was 6506. We excluded participants with unmatched age and sex (*n* = 289), those aged < 65 years (*n* = 3961), and those who were hospitalized patients or nursing home residents (*n* = 92). Finally, 2164 participants were included in this study.

Table 1 presents the characteristics of the study participants. The mean age of participants was 74.0 ± 6.8 years (mean ± standard deviation), and 1134 were women (52.4%). Of these, 456 (21.1%) reported EOH once or more per week. The median (interquartile range) number of teeth was 20 (range: 10–26). The percentage and numbers of EOH once or more per week with ≥20 teeth, 10–19 teeth, 1–9 teeth, and 0 teeth were 24.1% (*n* = 275), 21.5% (*n* = 104), 15.7% (*n* = 50), and 12.2% (*n* = 27), respectively. The variables associated with a higher frequency of EOH (≥once/week) were many teeth, younger age, men, current smokers, higher educational attainment, and working. Details of the frequency of EOH and the number of missing values before multiple imputation are shown in Supplementary Table S2. The percentage and numbers of frequency of EOH were never: 35.6% (*n* = 772), <once/week: 42.4% (*n* = 901), ≥once/week: 19.0% (*n* = 412), and ≥once/day: 1.6% (*n* = 35), respectively.

The results of the Poisson regression analysis of the association between the number of teeth and frequency of EOH are shown in Table 2. In Model 1, the crude model, a l number of teeth was significantly associated with a lower prevalence of EOH once or more per week compared to those with ≥20 teeth (PR for 10–19 teeth = 0.89, 95% CI: 0.73–1.09, PR for 1–9 teeth = 0.65, 95% CI: 0.49–0.86, and PR for 0 teeth = 0.51, 95% CI: 0.35–0.73, respectively). In Model 2, the sex- and age-adjusted model, a lower number of teeth was significantly associated with a lower prevalence of EOH once or more per week compared to those with ≥20 teeth (PR for 10–19 teeth = 0.90, 95% CI: 0.74–1.10, PR for 1–9 teeth = 0.68, 95% CI: 0.52–0.91, and PR for 0 teeth = 0.54, 95% CI: 0.37–0.79, respectively). In Model 3, adjusted for all covariates, a lower number of teeth was significantly associated with a lower prevalence of EOH once or more per week compared to those with ≥20 teeth (PR for 10–19 teeth = 0.89, 95% CI: 0.72–1.09, PR for 1–9 teeth = 0.67, 95% CI: 0.51–0.89, and PR for 0 teeth = 0.53, 95% CI: 0.36–0.77, respectively). When using the continuous variable of the number of teeth in a fully adjusted model, individuals with fewer teeth had a lower frequency of EOH than those with 32 teeth (*p*-for trend < 0.001), as shown in Supplementary Table S3.

In the sensitivity analysis of the complete case analysis, we observed an estimate similar to that of the main results (Supplementary Table S4). Supplementary Table S5 shows the characteristics of the study participants stratified by sex. There was a significant association between the number of teeth and the frequency of EOH only in men; however, a similar trend was observed in women (Supplementary Table S6). The interaction between the number of teeth and sex was not statistically significant, as shown in Supplementary Table S7. In addition, when we conducted a sensitivity analysis to assess how restaurant density affected each participant’s behavior, we found that the results were consistent with our main findings, as shown in Supplementary Table S8.

## 4. Discussion

We demonstrated an association between fewer teeth and a lower frequency of EOH among community-dwelling older adults in Japan. Although a few previous studies are directly related to our study, our findings are consistent with those of previous studies that have clarified the relationships between oral health, swallowing function, and food selection at home. Having fewer teeth may affect food choices because it causes difficulties in forming bolus, which then interferes with swallowing [42,43,44]. Being edentulous is a risk factor for changing food selections [45], and poor oral health is a risk factor for difficulty consuming 16 types of common foods [20]. Therefore, older adults with fewer teeth may face restrictions in food selection, not only at home but also at restaurants, which may lead them to avoid EOH.

One of the possible mechanisms by which fewer teeth may lead to reduced EOH behavior is the influence of SES. Fewer teeth are associated with a lower SES [46]. Furthermore, a lower SES results in decreased EOH behavior [28]. Thus, we examined the association between the number of teeth and the frequency of EOH while including SES in the model. Interestingly, even when the effect of SES on EOH was considered, the association remained robust. There may be pathways through which the number of teeth affects EOH behavior but not SES.

There is another possible pathway through which fewer teeth may reduce EOH. Having fewer teeth can decrease aesthetics, which is known to induce social isolation [21]. Over a long period, the reduction in the number of teeth might similarly lead to a gradual decrease in going out. For instance, broader social participation, including going out, has been suggested to decrease with fewer teeth in older adults [47]. With a reduction in outdoor activities, EOH may also decrease. Additionally, past EOH behaviors may adversely affect current oral health. The nutritional balance of most meals offered by restaurant establishments is unbalanced [5], and unhealthy meals have been suggested as a risk factor for tooth loss [48]. Therefore, individuals with fewer teeth and a lower frequency of EOH may have eaten out more frequently in the past.

The results of sensitivity analysis stratified by sex indicated that a significant association was observed only in men. Additionally, the interaction between the number of teeth and sex was not statistically significant (Supplementary Table S7). These results suggest that sex and the number of teeth independently affect EOH. Being a woman increases social participation in activities such as going out [49]. In addition, it has been suggested as an aspect of social participation [5]. In women with fewer teeth, EOH may still be part of social participation, such as meeting friends. Thus, women may maintain their EOH behaviors, even with fewer teeth, by going out to meet friends.

This study has an implication. It is important to promote oral health and improve the food environment and accessibility. To achieve Sustainable Development Goals (SDGs) Target 2.1 [50], which aims to increase the availability and access to nutritious foods that constitute a healthy diet, the government may support grocery stores in expanding their fresh food selections and restaurants by offering dishes that are rich in fruits and vegetables as healthier options. Although public health development is welcomed, many healthy foods and meals may not be suitable for older adults with poor oral health [51]. Providing services such as recipes that can be easily prepared and cooking classes is important to consume more food among older adults with poor oral health. A previous study indicated that cooking skills among older adults could be a modifiable predictor of mortality risk due to poor oral health [52]. Therefore, it is necessary to develop a food environment in accordance with the oral health perspective for community-dwelling older adults with poor oral health so that they can access foods and meals that they can chew and swallow.

The strength of this study lies in its use of statistical data from the Japanese government. The representativeness of our findings in Japan was confirmed using a large sample size. Our study has some limitations. First, it was impossible to clarify the causality because this was a cross-sectional study. For example, we did not consider the impact of past EOH behavior on the number of current teeth or EOH behavior. Alternatively, we cannot deny the possibility of reverse causality; that is, dietary habits in younger years could be a risk factor for tooth loss. Therefore, future studies using longitudinal data are warranted. Second, we adjusted for possible confounders in the association between the number of teeth and EOH. However, our study may have had unknown and residual confounders. Our data did not include information on dental prosthetics such as denture use. A previous study suggested that older adults with fewer teeth and those who use dentures consume more protein than those with fewer teeth and those who do not use dentures [18]. In addition, a previous study reported that having fewer teeth was associated with a higher prevalence of eating alone and that dental prosthesis use reduced this association [53]. Therefore, older adults who have fewer teeth with dentures may exhibit more functional and social aspects than those who have fewer teeth without dentures, and our findings may have been underestimated. However, the association between number of teeth and low EOH remained robust. Further studies are required to determine the association between the consumption of more protein and more frequent EOH. The data used in this study lacked some SES variables, such as income and property of older adults, which are often considered SES variables in previous studies [54]. Although we accounted for the impact of SES by including educational attainment and employment status in the model, the unmeasured effects of these SES factors may have influenced the point estimates. Therefore, caution is required when interpreting the results. Furthermore, we did not consider geographical access to EOH. Some participants may have lived in areas with few or no eating out places, such as restaurants, affecting their EOH behavior. To address this issue, we included city-level data as a surrogate for restaurant density in our model. These results were consistent with those of the main analysis (Supplementary Table S8). There were other unmeasured confounders in addition to dental prosthesis use and geographical access. For example, our data do not include information on food access via delivery from restaurants and grocery stores. Therefore, among those with less frequent EOH, a certain number may consume meals provided through delivery services. However, it is presumed that access to food via delivery, which involves additional charges in addition to the product price, is strongly influenced by SES and the availability of delivery services in urban areas. In our study, the association between fewer teeth and less frequent EOH was consistently observed in the results of the sensitivity analysis, including SES and urban residence as covariates (Supplementary Table S8). Therefore, the effect of delivery services on our results was limited. It is necessary to clarify which methods of food access affect healthy dietary intake among older adults with fewer teeth. Future analyses using survey data, including detailed questions on EOH and related confounders, are required. Third, the generalizability of our study may be limited because of variations in the definition, concept, and culture of “EOH” across countries [5]. For example, in a study from Greece, although limited by a small sample size, the impact of oral health on the frequency of eating out was limited [55]. Further studies conducted in multiple countries are required.

## 5. Conclusions

Having fewer teeth was associated with lower EOH behavior in older adults. This association might be explained by multiple pathways, such as difficulties in mastication and reduced social interaction. The findings suggest that preventing tooth loss could help maintain food accessibility for older adults by supporting their EOH behavior. Future research should focus on longitudinal studies to establish causal relationships and develop targeted interventions.

## Figures and Tables

**Table 1:** Characteristics of the study participants after multiple imputation (*n* = 2164).

	Total (*n* = 2164)		Frequency of EOH			
			<Once/Week (*n* = 1708; 78.9%)		≥Once/Week (*n* = 456; 21.1%)	
	*n*	% ^a^	*n*	% ^b^	*n*	% ^b^
**Number of teeth**						
≥20 teeth	1142	52.8	867	75.9	275	24.1
10–19 teeth	483	22.3	379	78.5	104	21.5
1–9 teeth	318	14.7	268	84.3	50	15.7
0 teeth	221	10.2	194	87.8	27	12.2
**Age**						
65–69	658	30.4	489	74.3	169	25.7
70–74	596	27.5	473	79.4	123	20.60
75–79	447	20.7	357	79.9	90	20.1
80–84	273	12.6	223	81.7	50	18.3
≥85	190	8.8	166	87.5	24	12.5
**Sex**						
Men	1030	47.6	759	73.7	271	26.3
Women	1134	52.4	949	83.7	185	16.3
**Smoking status**						
Never	1748	80.8	1404	80.3	344	19.7
Quit	166	7.7	125	75.5	41	24.5
Current	250	11.6	179	71.5	71	28.5
**Drinking habits**						
Never	1263	58.4	1022	80.9	241	19.1
Quit	63	2.9	53	83.8	10	16.2
Current	838	38.7	633	75.6	205	24.4
**Educational attainment**						
≤9 years	510	23.6	424	83.0	87	17.0
10–12 years	1089	50.3	868	79.8	220	20.2
≥13 years	565	26.1	416	73.7	149	26.3
**Living arrangement**						
Living with others	1801	83.2	1430	79.4	371	20.6
Living alone	363	16.8	278	76.6	85	23.4
**Marital status**						
Married	1572	72.6	1244	79.2	328	20.8
Not married	91	4.2	67	73.2	24	26.8
Divorced/widowed	501	23.2	397	79.3	104	20.7
**Working status**						
Not working	1512	69.9	1237	81.8	275	18.2
Working	652	30.1	471	72.3	181	27.7
**Psychological distress**						
Low ^1^	1600	74.0	1257	78.6	343	21.4
High ^2^	564	26.0	451	79.9	113	20.1
**Medical institution visits**						
Yes	1581	73.1	1260	79.7	321	20.3
No	583	26.9	448	76.8	135	23.2
**Subjective health status**						
Good	511	23.6	393	77.0	118	23.0
Normal	1171	54.1	926	79.1	245	20.9
Bad	482	22.3	389	80.7	93	19.3
**Healthy food intake**						
No	1529	70.7	1208	79.0	321	21.0
Yes	635	29.3	500	78.8	135	21.2
**Social participation**						
Yes	1395	64.5	1097	78.6	298	21.4
No	769	35.5	611	79.5	158	20.5
**Population size**						
Metropolitan area	367	17.0	262	71.4	105	28.6
≥150,000	683	31.6	517	75.7	166	24.3
<150,000	1114	51.5	929	83.4	185	16.6

Note: EOH, eating out of home. ^1^ Low psychological distress means total K6 score < 5. ^2^ High psychological distress means total K6 score ≥ 5. ^a^ = This percentage shows the col %. ^b^ = This percentage shows the row %.

**Table 2:** Association between the number of teeth and the frequency of EOH (*n* = 2164).

	Model 1 ^a^		Model 2 ^b^		Model 3 ^c^	
	PR †	(95% CI)	PR †	(95% CI)	PR †	(95% CI)
**Number of teeth**						
≥20 teeth	1.00	(Ref.)	1.00	(Ref.)	1.00	(Ref.)
10–19 teeth	0.89	(0.73–1.09)	0.90	(0.74–1.10)	0.89	(0.72–1.09)
1–9 teeth	0.65	(0.49–0.86) **	0.68	(0.52–0.91) **	0.67	(0.51–0.89) **
0 teeth	0.51	(0.35–0.73) ***	0.54	(0.37–0.79) **	0.53	(0.36–0.77) **

Note: EOH, eating out of home; PR, prevalence ratio; CI, confidence intervals; Ref, reference. ** *p* < 0.01; *** *p* < 0.001. † PR was estimated by a modified Poisson regression model with all variables simultaneously entered in the model. ^a^ = Model 1 was the crude model. ^b^ = Model 2 was adjusted for age and sex. ^c^ = Model 3 was adjusted for age, sex, smoking status, drinking habits, educational attainment, living arrangement, marital and working status, psychological distress, medical institution visits, subjective health status, healthy food intake, and social participation.

## Data Availability

The data we used in this study are available from the Ministry of Health, Labour and Welfare in Japan for researchers who meet the criteria established under the Statistics Act.

## Supplementary Materials

The following supporting information can be downloaded at: https://www.mdpi.com/article/10.3390/nu16132102/s1, Supplementary Table S1: Details of covariates questionnaire items; Supplementary Table S2: Characteristics of study participants (the frequency of EOH in four categories) including missing values; Supplementary Table S3: Association between the number of teeth (continuous variable) and the frequency of EOH (*n* = 2164); Supplementary Table S4: Association between the number of teeth and the frequency of EOH in complete case analysis (*n* = 1514); Supplementary Table S5: Characteristics of the study participants according to sex; Supplementary Table S6: Association between the number of teeth and the frequency of EOH according to sex; Supplementary Table S7: The interaction between sex and number of teeth on the probability of EOH (*n* = 2164); Supplementary Table S8: Association between the number of teeth and the frequency of EOH (additional adjusted for the city-level variable) (*n* = 2164).
