# Supplementary material for: Association between Number of Teeth and Eating out of Home: A 2019 Statistical Survey of the Japanese Representative Population
Source: Nutrients. 2024 Jul 1;16(13):2102. doi: 10.3390/nu16132102 (PMC11243636; doi:10.3390/nu16132102)
Supplement: Supplementary file 1 [file nutrients-16-02102-s001.zip › nutrients-3066876-supplementary.pdf]

Title: Association between Number of Teeth and Eating Out of Home: A 2019 Statistical Survey on the Japanese Representative Population

Table of Contents

Supplementary Table S1. Details of covariates questionnaire items 2

Supplementary Table S2. Characteristics of study participants (the frequency of EOH in four categories) including missing values 4

Supplementary Table S3. Association between the number of teeth (continuous variable) and the frequency of EOH (n=2,164) 7

Supplementary Table S4. Association between the number of teeth and the frequency of EOH in complete case analysis (n=1,514) 8

Supplementary Table S5. Characteristics of the study participants according to sex 9

Supplementary Table S6. Association between the number of teeth and the frequency of EOH according to sex 11

Supplementary Table S7. The interaction between sex and number of teeth on the probability of EOH (n=2,164) 11

Supplementary Table S8. Association between the number of teeth and the frequency of EOH (additional adjusted for the city-level variable) (n=2,164) 13

Supplementary Table S1. Details of covariates questionnaire items

	Question text	Answer choices
Smoking status	Do you smoke?	1: I smoke every day.<br>2: I smoke sometimes.<br>3: I used to smoke, but I have not smoked for over a month now.<br>4: Never.
Drinking habits	How many days per week do you drink alcohol (sake, shochu, beer, western liquor, etc.)?	1: Every day.<br>2: 5–6 days a week.<br>3: 3–4 days a week.<br>4: 1–2 days a week.<br>5: 1–3 days a month.<br>6: I hardly drink.<br>7: Quit.<br>8: Never.
Educational attainment	Are you currently enrolled in school?	**Status**<br>1: Currently enrolled.<br>2: Graduated.<br>3: Never.<br>**Type of school**<br>A: Elementary school/junior high school.<br>B: High school/old system junior high school.<br>C: Vocational school.<br>D: Junior college/technical college.<br>E: University.<br>D: Graduate school.
Marital status	What is your marital status?	1: Married.<br>2: Not married.<br>3: Widow.<br>4: Divorce
Working status	Please select your employment status in May (the past month).	1: Mainly working.<br>2: Mainly housework and working.<br>3: Mainly commuting to school and working.<br>4: Other (working).<br>5: Commuting to school (not working).<br>6: Housework (not working).<br>7: Other (not working).
Psychological distress	During a past month, about how often did you about six items?<br>1: Feeling nervous.<br>2: Feeling hopeless.<br>3: Feeling restless or fidgety.<br>4: Feeling so depressed that nothing could cheer you up.<br>5: Feeling that everything was an effort.<br>6: Feeling worthless.	1: Always.<br>2: Usually.<br>3: Sometimes.<br>4: Little.<br>5: Never.

Medical institution visits	Do you currently go to a hospital, clinic (medical/dental), massage, acupuncture, or treatment center due to injury or illness?	1: Yes.<br>2: No.
Subjective health status	How is your current health condition?	1: Excellent.<br>2: Good.<br>3: Normal.<br>4: Fair.<br>5: Poor.
Healthy food intake	Do you use healthy food (including nutrients that to keep or improve your health, whether in pill, capsule, powder, or liquid form), such as nutritional supplements?	1: Yes.<br>2: No.
Social participation	Do you participate in group activities such as neighborhood associations, community events, volunteer activities, sports, or hobbies?<br>What activities do you participate in and how often?	**Activities**<br>A: Neighborhood associations, community events.<br>B: Volunteer activities.<br>C: Sports.<br>D: Hobbies.<br>E: Other.<br>**Frequency**<br>1: ≥4 times a week.<br>2: 2–3 times a week.<br>3: Onece a week.<br>4: 1–3 times a month.<br>5: Sometimes a year.<br>6: No participate.

Living arrangement were composed of the household number and the relationship with the head of the household.

Supplementary Table S2. Characteristics of study participants (the frequency of EOH in four categories) including missing values

	Total (n = 2,164)		Frequency of EOH									
			Never (n = 772)		<1 time/week (n = 901)		≥1 time/week (n = 412)		≥1 time/day (n = 35)		Missing (n = 44)	
	n	%[a]	n	%[b]	n	%[b]	n	%[b]	n	%[b]	n	%[b]
**Number of teeth**												
≥20 teeth	1,112	51.4	346	31.1	498	44.8	249	22.4	19	1.7	0	0.0
10-19 teeth	461	21.3	170	36.9	190	41.2	92	20.0	8	1.7	1	0.2
1-9 teeth	307	14.2	134	43.6	125	40.7	43	14.0	5	1.6	0	0.0
0 teeth	221	10.2	113	51.1	81	36.7	24	10.9	3	1.4	0	0.0
Missing	63	2.9	9	14.3	7	11.1	4	6.3	0	0.0	43	68.3
**Age**												
65–69	658	30.4	176	26.7	305	46.4	148	22.5	19	2.9	10	1.5
70–74	596	27.5	201	33.7	262	44.0	114	19.1	6	1.0	13	2.2
75–79	447	20.7	173	38.7	177	39.6	80	17.9	8	1.8	9	2.0
80–84	273	12.6	108	39.6	111	40.7	47	17.2	2	0.7	5	1.8
≥85	190	8.8	114	60.0	46	24.2	23	12.1	0	0.0	7	3.7
**Sex**												
Men	1,030	47.6	352	34.2	395	38.3	240	23.3	27	2.6	16	1.6
Women	1,134	52.4	420	37.0	506	44.6	172	15.2	8	0.7	28	2.5
**Smoking status**												
Never	1,710	79.0	636	37.2	736	43.0	312	18.2	25	1.5	1	0.1
Quit	163	7.5	45	27.6	78	47.9	39	23.9	1	0.6	0	0.0
Current	245	11.3	90	36.7	85	34.7	61	24.9	9	3.7	0	0.0
Missing	46	2.1	1	2.2	2	4.3	0	0.0	0	0.0	43	93.5
**Drinking habits**												
Never	1,234	57.0	481	39.0	516	41.8	219	17.7	17	1.4	1	0.1
Quit	62	2.9	31	50.0	21	33.9	9	14.5	1	1.6	0	0.0
Current	824	38.1	259	31.4	364	44.2	184	22.3	17	2.1	0	0.0
Missing	44	2.0	1	2.3	0	0.0	0	0.0	0	0.0	43	97.7
**Educational attainment**												
≤9 years	410	18.9	186	45.4	145	35.4	63	15.4	4	1.0	12	2.9
10-12 years	886	40.9	334	37.7	360	40.6	160	18.1	12	1.4	20	2.3
≥13 years	462	21.3	114	24.7	226	48.9	110	23.8	12	2.6	0	0.0

Missing	406	18.8	138	34.0	170	41.9	79	19.5	7	1.7	12	3.0
**Living arrangement**												
Living with others	1,801	83.2	637	35.4	763	42.4	336	18.7	27	1.5	38	2.1
Living alone	363	16.8	135	37.2	138	38.0	76	20.9	8	2.2	6	1.7
**Marital status**												
Married	1,572	72.6	536	34.1	688	43.8	296	18.8	26	1.7	26	1.7
Not married	91	4.2	29	31.9	36	39.6	22	24.2	2	2.2	2	2.2
Divorced/widowed	501	23.2	207	41.3	177	35.3	94	18.8	7	1.4	16	3.2
**Working status**												
Not working	1,442	66.6	550	38.1	605	42.0	241	16.7	15	1.0	31	2.1
Working	626	28.9	186	29.7	259	41.4	155	24.8	18	2.9	8	1.3
Missing	96	4.4	36	37.5	37	38.5	16	16.7	2	2.1	5	5.2
**Psychological distress**												
Low[1]	1,481	68.4	511	34.5	624	42.1	295	19.9	23	1.6	28	1.9
High[2]	509	23.5	192	37.7	203	39.9	92	18.1	9	1.8	13	2.6
Missing	174	8.0	69	39.7	74	42.5	25	14.4	3	1.7	3	1.7
**Medical institution visits**												
Yes	1,564	72.3	567	36.3	656	41.9	286	18.3	26	1.7	29	1.9
No	577	26.7	199	34.5	233	40.4	123	21.3	8	1.4	14	2.4
Missing	23	1.1	6	26.1	12	52.2	3	13.0	1	4.3	1	4.3
**Subjective health status**												
Good	504	23.3	172	34.1	209	41.5	111	22.0	4	0.8	8	1.6
Normal	1,158	53.5	405	35.0	491	42.4	216	18.7	23	2.0	23	2.0
Bad	475	22.0	185	38.9	188	39.6	83	17.5	6	1.3	13	2.7
Missing	27	1.2	10	37.0	13	48.1	2	7.4	2	7.4	0	0.0
**Healthy food intake**												
No	1,497	69.2	537	35.9	618	41.3	289	19.3	20	1.3	33	2.2
Yes	621	28.7	218	35.1	263	42.4	118	19.0	13	2.1	9	1.4
Missing	46	2.1	17	37.0	20	43.5	5	10.9	2	4.3	2	4.3
**Social participation**												
Yes	1,364	63.0	439	32.2	634	46.5	275	20.2	15	1.1	1	0.1
No	750	34.7	331	44.1	266	35.5	134	17.9	19	2.5	0	0.0
Missing	50	2.3	2	4.0	1	2.0	3	6.0	1	2.0	43	86.0
**Population size**												
Metropolitan area	367	17.0	109	29.7	146	39.8	96	26.2	7	1.9	9	2.5

≥150,000	683	31.6	236	34.6	274	40.1	145	21.2	18	2.6	10	1.5
<150,000	1,114	51.5	427	38.3	481	43.2	171	15.4	10	0.9	25	2.2

Note: EOH, Eating Out of Home

[1] Low psychological distress means total K6 score <5.

[2] High psychological distress means total K6 score ≥5.

a = This percentage shows the col %.

b = This percentage shows the row %.

Supplementary Table S3. Association between the number of teeth (continuous variable) and the frequency of EOH (n=2,164)

	Model 1[a]		Model 2[b]		Model 3[c]	
	PR†	(95% CI)	PR†	(95% CI)	PR†	(95% CI)
**Number of teeth**						
Continuous variable	0.97	(0.97–0.98)***	0.98	(0.97–0.99)***	0.98	(0.97–0.99)***

Note: EOH, Eating Out of Home; PR, Prevalence Ratio; CI, Confidence Intervals.

*p < 0.05; **p < 0.01; ***p < 0.001

†PR was estimated by a modified Poisson regression model with all variables simultaneously entered the model.

a = Model 1 was crude model.

b = Model 2 adjusted for age and sex.

c = Model 3 adjusted for age, sex, smoking status, drinking habits, educational attainment, living arrangement, marital and working status, psychological distress, medical institution visits, subjective health status, healthy food intake, and social participation.

Supplementary Table S4. Association between the number of teeth and the frequency of EOH in complete case analysis (n=1,514)

	Model 1[a]		Model 2[b]		Model 3[c]	
	PR†	(95% CI)	PR†	(95% CI)	PR†	(95% CI)
**Number of teeth**						
≥20 teeth	1.00	(Ref.)	1.00	(Ref.)	1.00	(Ref.)
10-19 teeth	0.97	(0.77–1.22)	0.98	(0.78–1.23)	0.97	(0.77–1.22)
1-9 teeth	0.56	(0.39–0.80)**	0.59	(0.41–0.86)**	0.58	(0.40–0.84)**
0 teeth	0.55	(0.36–0.85)**	0.59	(0.38–0.91)*	0.58	(0.38–0.90)*

Note: EOH, Eating Out of Home; PR, Prevalence Ratio; CI, Confidence Intervals.

*p < 0.05; **p < 0.01; ***p < 0.001

†PR was estimated by a modified Poisson regression model with all variables simultaneously entered the model.

a = Model 1 was crude model.

b = Model 2 adjusted for age and sex.

c = Model 3 adjusted for age, sex, smoking status, drinking habits, educational attainment, living arrangement, marital and working status, psychological distress, medical institution visits, subjective health status, healthy food intake, and social participation.

Supplementary Table S5. Characteristics of the study participants according to sex

	Men		Women	
	Total (n = 1,030) n	≥ once/week of EOH (n = 271) n (%)	Total (n = 1,134)	≥ once/week of EOH (n = 185) n (%)
**Number of teeth**				
≥20 teeth	524	165 (31.5)	618	110 (17.8)
10-19 teeth	242	64 (26.3)	241	40 (16.6)
1-9 teeth	153	26 (16.9)	165	24 (14.6)
0 teeth	111	16 (14.4)	110	11 (10.0)
**Age**				
65–69	335	119 (35.5)	323	51 (15.6)
70–74	282	72 (25.5)	314	52 (16.8)
75–79	220	46 (21.0)	227	44 (19.2)
80–84	121	25 (20.9)	152	25 (16.3)
≥85	72	9 (12.6)	118	15 (12.5)
**Smoking status**				
Never	683	174 (25.5)	1,064	170 (15.9)
Quit	150	38 (25.3)	17	3 (18.9)
Current	197	59 (30.0)	53	12 (23.1)
**Drinking habits**				
Never	358	96 (26.8)	905	146 (16.1)
Quit	50	7 (14.4)	14	3 (23.1)
Current	622	168 (27.0)	215	36 (16.7)
**Educational attainment**				
≤9 years	196	39 (19.6)	314	48 (15.3)
10-12 years	503	123 (24.5)	586	97 (16.6)
≥13 years	331	109 (33.0)	234	40 (16.9)
**Living arrangement**				
Living with others	903	219 (24.3)	898	152 (16.9)
Living alone	127	52 (40.6)	236	33 (14.2)
**Marital status**				
Married	866	214 (24.7)	706	114 (16.1)
Not married	48	20 (42.3)	43	4 (9.4)
Divorced/widowed	116	37 (31.6)	385	67 (17.4)
**Working status**				
Not working	610	131 (21.5)	902	144 (16.0)
Working	420	140 (33.3)	232	41 (17.6)
**Psychological distress**				
Low[1]	771	204 (26.4)	829	139 (16.7)
High[2]	259	67 (25.8)	305	46 (15.2)
**Medical institution visits**				
Yes	754	189 (25.1)	827	132 (16.0)
No	276	82 (29.8)	307	53 (17.2)
**Subjective health status**				
Good	265	82 (30.9)	246	36 (14.7)
Normal	550	135 (24.6)	621	110 (17.6)
Bad	215	54 (25.1)	267	39 (14.7)
**Healthy food intake**				
No	744	194 (26.1)	785	127 (16.2)

Yes	286	77 (26.8)	349	58 (16.6)
**Social participation**				
Yes	660	172 (26.1)	735	126 (17.2)
No	370	99 (26.8)	399	59 (14.7)

Note: EOH, Eating Out of Home

[1] Low psychological distress means total K6 score <5.

[2] High psychological distress means total K6 score ≥5.

Supplementary Table S6. Association between the number of teeth and the frequency of EOH according to sex

	Men (n = 1,030)				Women (n = 1,134)			
	Model 1[a]		Model 2[b]		Model 1[a]		Model 2[b]	
	PR†	(95% CI)	PR†	(95% CI)	PR†	(95% CI)	PR†	(95% CI)
**Number of teeth**								
≥20 teeth	1.00	(Ref.)	1.00	(Ref.)	1	(Ref.)	1	(Ref.)
10-19 teeth	0.83	(0.65–1.07)	0.88	(0.68–1.13)	0.93	(0.67–1.31)	0.89	(0.63–1.26)
1-9 teeth	0.53	(0.37–0.78)**	0.59	(0.41–0.86)**	0.82	(0.54–1.24)	0.81	(0.53–1.22)
0 teeth	0.46	(0.28–0.73)**	0.51	(0.32–0.81)**	0.56	(0.31–1.01)	0.56	(0.31–1.01)

Note: EOH, Eating Out of Home; PR, Prevalence Ratio; CI, Confidence Intervals.
*p < 0.05; **p < 0.01; ***p < 0.001
†PR was estimated by a modified Poisson regression model with all variables simultaneously entered the model.
a = Model 1 was crude model.
b = Model 2 adjusted for age, smoking status, drinking habits, educational attainment, living arrangement, marital and working status, psychological distress, medical institution visits, subjective health status, healthy food intake, and social participation.

Supplementary Table S7. The interaction between sex and number of teeth on the probability of EOH (n=2,164)

	PR (95% CI)	*p*-value
**Mein effect: Number of teeth**		
≥20 teeth	1.00	(Ref.)
10-19 teeth	0.84 (0.66–1.08)	0.186
1-9 teeth	0.57 (0.39–0.82)**	0.003
0 teeth	0.49 (0.31–0.78)**	0.003
**Interaction effect†**		
10-19 teeth # Women	1.13 (0.74–1.72)	0.562
1-9 teeth # Women	1.49 (0.85–2.61)	0.165
0 teeth # Women	1.24 (0.59–2.60)	0.572

Note: EOH, Eating Out of Home; PR, Prevalence Ratio; CI, Confidence Intervals.
*p < 0.05; **p < 0.01; ***p < 0.001

† p-value of the linear trend of the interaction (p-for trend) was 0.190.
Adjusted for age, sex, smoking status, drinking habits, educational attainment, living arrangement, marital and working status, psychological distress, medical institution visits, subjective health status, healthy food intake, and social participation.

Supplementary Table S8. Association between the number of teeth and the frequency of EOH (additional adjusted for the city-level variable) (n=2,164)

	PR†	(95% CI)
**Number of teeth**		
≥20 teeth	1.00	(Ref.)
10-19 teeth	0.91	(0.74–1.12)
1-9 teeth	0.69	(0.52–0.91)**
0 teeth	0.55	(0.38–0.80)**

Note: EOH, Eating Out of Home; PR, Prevalence Ratio; CI, Confidence Intervals; Ref.. Reference.

*p < 0.05; **p < 0.01; ***p < 0.001

†PR was estimated by a modified Poisson regression model with all variables simultaneously entered the model.

Adjusted for age, sex, smoking status, drinking habits, educational attainment, living arrangement, marital and working status, psychological distress, medical institution visits, subjective health status, healthy food intake, social participation, and city-level variable.
